# Regional chemotherapy by isolated limb perfusion prior to surgery compared with surgery and post-operative radiotherapy for primary, locally advanced extremity sarcoma: a comparison of matched cohorts

**DOI:** 10.1186/s13569-018-0098-6

**Published:** 2018-07-02

**Authors:** Jens Jakob, Henry G. Smith, Michelle J. Wilkinson, Tim Pencavel, Aisha B. Miah, Joseph M. Thomas, Per-Ulf Tunn, Lothar R. Pilz, Dirk C. Strauss, Peter Hohenberger, Andrew J. Hayes

**Affiliations:** 1 Department of Surgery, Division of Surgical Oncology, University Hospital Mannheim, University of Heidelberg, Mannheim, Germany; 2 Sarcoma Unit, Department of Academic Surgery, Royal Marsden Hospital NHS Foundation Trust, Fulham Road, London, SW3 6JJ UK; 3 Department of Orthopedic Oncology, Helios Klinikum Berlin-Buch, Sarcoma Center Berlin-Brandenburg, Berlin, Germany; 4 Medical Faculty Mannheim, University of Heidelberg, Mannheim, Germany; 5 Department of Surgery, Division of Surgical Oncology and Thoracic Surgery, University Hospital Mannheim, University of Heidelberg, Mannheim, Germany

**Keywords:** Soft tissue sarcoma, Isolated limb perfusion, Adjuvant radiotherapy, Postoperative complications

## Abstract

**Background:**

Induction chemotherapy by isolated limb perfusion (ILP) with melphalan and tumour necrosis factor-α is an effective strategy to facilitate limb-conserving surgery in locally advanced extremity sarcoma. In a comparison of cohorts matched for grade, size and surgical resectability, we compared the outcome of patients undergoing induction ILP prior to limb-conserving surgery and selective post-operative radiotherapy with patients undergoing limb-conserving surgery and routine post-operative radiotherapy.

**Methods:**

Patients with primary, grade 2/3 sarcomas of the lower limbs over 10 cm in size were identified from prospectively maintained databases at 3 centres. Patients treated at a UK centre underwent limb-conserving surgery and post-operative radiotherapy (Standard cohort). Patients at two German centres underwent induction ILP, limb-conserving surgery and selective post-operative radiotherapy (ILP cohort).

**Results:**

The Standard cohort comprised 80 patients and the ILP cohort 44 patients. Both cohorts were closely matched in terms of tumour size, grade, histological subtype and surgical resectability. The median age was greater in the Standard vs the ILP cohort (60.5 years vs 56 years, p = 0.033). The median size was 13 cm in both cohorts. 5-year local-recurrence (ILP 12.2%, Standard 20.1%, p = 0.375) and distant metastases-free survival rates (ILP 49.6%, Standard 46.0% p = 0.821) did not differ significantly between cohorts. Fewer patients received post-operative radiotherapy in the ILP cohort compared with the Standard cohort (27% vs 82%, p < 0.001).

**Conclusion:**

In comparative cohorts, the outcomes of patients undergoing induction ILP prior to surgery did not differ from those undergoing standard management, although induction ILP was associated with a reduced need for adjuvant radiation.

**Electronic supplementary material:**

The online version of this article (10.1186/s13569-018-0098-6) contains supplementary material, which is available to authorized users.

## Background

Extremity soft tissue sarcomas (ESTS) are rare tumours comprising over 50 different histological subtypes [1, 2]. Optimal management of locally advanced ESTS requires multimodal therapy and the precise role of isolated limb perfusion (ILP) in the overall treatment strategy remains to be fully defined. For small or superficial ESTS, treatment usually involves surgery alone, gaining wide surgical margins while preserving function. Adjuvant radiotherapy is reserved for high-grade tumours greater than 5 cm or small tumours that focally involve margins adjacent to a critical structure [3]. When sarcomas attain even greater dimensions, such as over 10 cm, it becomes increasingly difficult to achieve negative margins without recourse to amputation or function-limiting surgery. While amputation for ESTS does not improve survival for sarcoma over limb-conserving surgery, it may still be considered for large sarcomas when the risk for local recurrence is viewed as significant [4–6].

Induction chemotherapy by ILP using melphalan with recombinant human tumour necrosis factor alpha (TNFα) prior to a limb-conserving surgical resection was introduced as a strategy for locally advanced sarcomas considered irresectable other than by an amputation [7]. TNFα is a multifunctional cytokine, which causes increased vascular permeability, associated with increased extravasation of cytotoxic agents and selective destruction of tumour-associated vessels by endothelial apoptosis and inflammation [8–10]. A multi-institutional case series of patients with locally advanced sarcomas, considered to be irresectable with limb-conserving surgery and adjuvant radiotherapy, which were treated using ILP with TNFα reported a limb salvage rate of 84% [7]. In light of these results, TNFα has been licensed for this indication since 2006.

ILP is now used more widely in large, high-grade tumours that are compatible with limb-conserving surgery but risk positive resection margins. Such patients would undergo an induction ILP prior to a wide resection of the primary tumour, with the aim of gaining as wide a margin as is compatible with preserving limb function. Post-operative radiotherapy is then typically only offered if the pathology specimen demonstrates viable tumour at a compromised margin. The alternative, which might be considered the standard management, would be a function-preserving wide resection with adjuvant radiotherapy to compromised margins. It is known that with adjuvant radiotherapy, planned microscopic positive margins over a critical structure are fully compatible with long-term local control [11].

In a retrospective cohort analysis, we sought to determine whether the peri-operative morbidity and oncological outcomes differed between these alternative multi-modality approaches to large high-grade sarcoma of the lower limb.

## Patients and methods

### Patient selection

Patients were identified from 1996 to 2010 using prospectively maintained databases at the Royal Marsden Hospital London, UK (defined as the Standard cohort) and the Sarcoma Center Berlin-Brandenburg and the University Medical Center Mannheim, Germany (defined as the ILP cohort). In the ILP cohort, all patients with a primary, unifocal, intermediate or high-grade (G2 and G3) ESTS of the lower limb with a maximum dimension of over 10 cm who underwent an induction ILP prior to a surgical resection were included. In the Standard cohort, all patients with the same characteristics but who had undergone limb-conserving surgery were included. Patients with disseminated disease at diagnosis or who had received pre-operative systemic chemotherapy were excluded.

Grading was according to the FNCLCC system [12]. Each tumour was staged as T2b N0 MO G2 or G3, corresponding to stage IIb or III in the AJCC system [13]. Size was defined by pre-operative cross sectional imaging (CT or MRI) and confirmed after pathological analysis. The administration of adjuvant radiotherapy in either cohort was not an inclusion criterion and all decisions relating to radiotherapy were made on an individual patient basis.

### Treatment

The ILP cohort underwent induction ILP with TNFα (Beromun™, Boehringer Ingelheim, Germany) and melphalan followed by a wide or compartmental resection of the tumour 6–10 weeks later. Adjuvant radiation was not given routinely but considered in case of R1 resections or where the rate of necrosis post-ILP was considered suboptimal. The Standard cohort underwent a wide or compartmental resection, with radiotherapy offered at 6–12 weeks post-operatively [14].

### Isolated limb perfusion

ILP has been described in detail before [7]. The procedure was performed under general anaesthesia. The perfusions were hyperthermic, with a target temperature of 38–39.5 °C. TNFα was administered after a stable limb circuit without leakage had been established, at a dose of 2–4 mg. Melphalan was applied 15 min later, at a dose of 10 mg/L of perfused limb volume. Total perfusion time was 90 min. The extremity was then rinsed with hydroxyl ethyl starch.

### Surgical resection

The surgical approach in both cohorts was to achieve negative surgical histopathological margins where possible in the context of a limb-conserving operation. En-bloc resections with wide margins (1–2 cm of uninvolved tissue or an intact adjacent fascial layer) were performed whenever possible. When the tumour abutted major vessels or motor nerves, the adventitia or epineurium was taken as the margin of resection. When vessels were encased, they were resected and reconstructed. When the tumour abutted the bone, the periosteum was taken en-bloc with the tumour. Soft tissue reconstruction with pedicled or free-flaps was performed whenever necessary.

### Adjuvant radiotherapy

Post-operative radiotherapy was administered 6–10 weeks post-surgery. In the Standard cohort, post-operative radiotherapy was considered for all cases. However, the final decision was made by a multi-disciplinary team in light of post-operative histology, the surgical margins and the patient’s age and co-morbidities. In the ILP cohort, radiotherapy was administered in selected cases when there was a suboptimal histopathological response (less than 90% necrosis or viable tumour present at a surgical margin).

### Histopathological analysis

Histopathologic analysis included assessment of resection margins and pathological response. A macroscopically positive margin was defined as R2 resection. If the tumour extended into the resection margin (< 1 mm) on microscopic examination, the margin was defined as R1 resection and margins without actual involvement of the resection margin (> 1 mm or an intact fascial plane) were considered microscopically negative (R0).

### Follow-up

Patients were followed up every 3–4 month intervals for the first 3 years, then twice a year for up to 5 years, and annually thereafter.

### Cohort comparison for resectability

**Fig. 1:**
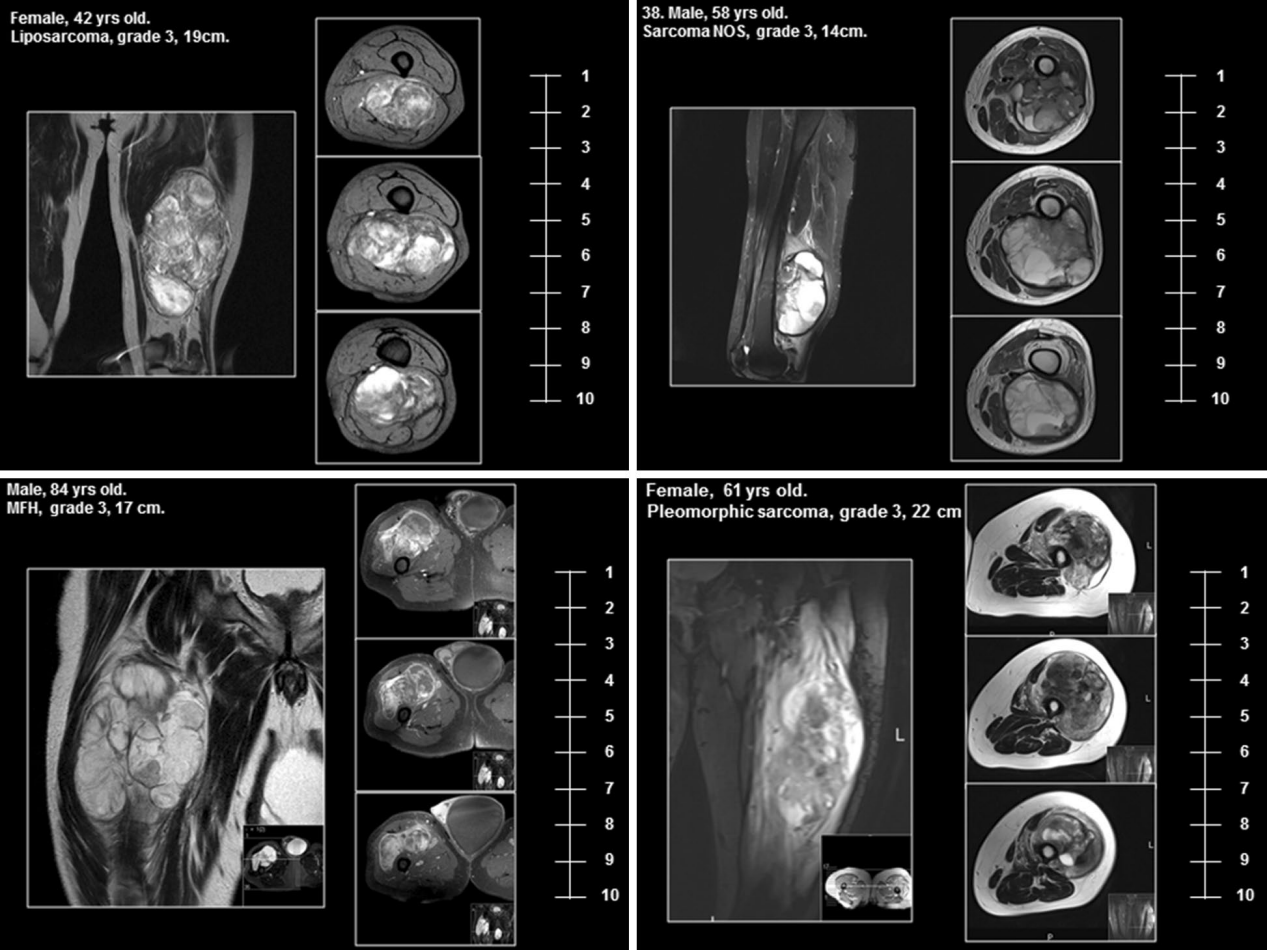
Examples of MRI imaging of patients used for comparison of tumour resectability in cohorts

To ensure that the tumours in both cohorts were equivalent in terms of resectability, three independent sarcoma surgeons working at major European sarcoma centres reviewed anonymised MRI’s from patients within each cohort and scored the compatibility of the tumours with limb-conserving surgery and adjuvant radiotherapy alone. Twenty patients were randomly selected from each cohort. The anonymised images, together with basic patient details (gender, age, histopathology and tumour size), were then distributed to the assessors who ranked the images based on resectability from 1 to 10, with 1 being easily resectable by limb-conserving surgery and 10 indicating that limb conservation was impossible and amputation was required (Fig. 1).

### Statistical analysis

To identify risk factors for local recurrence, metastasis and death from disease, stepwise Cox proportional-hazards regression analyses were performed using SPSS version 20. The influence of age (< 65 vs. ≥ 65 years), tumour size, tumour grade (2 vs. 3), resection margin (R0 vs. R1), post-operative irradiation (yes vs. no), and local recurrence were assessed. Overall survival, systemic and local recurrences were calculated using Kaplan–Meier method and compared using the logrank test (Graphpad Prism, Version 6.0).

## Results

### Patient characteristics

**Fig. 2:**
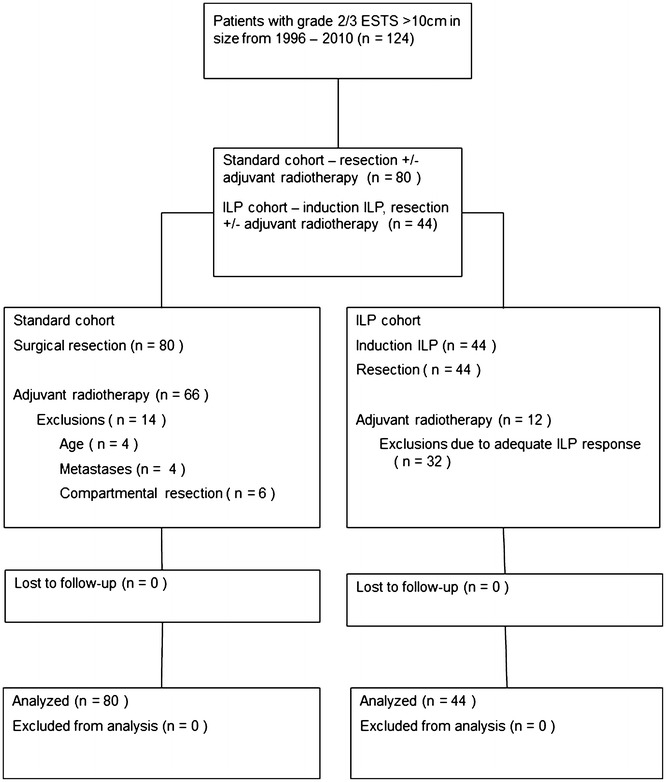
CONSORT diagram of the Standard and ILP cohorts

Details of patient demographics are shown in Table 1. 80 patients were identified in the Standard cohort and compared to 44 patients in the ILP cohort (Fig. 2). The median age of patients in the Standard cohort was 60.5 years (range 18–92), which was significantly older than the ILP cohort with a median age of 56 years (range 17–82), (Mann–Whitney test p = 0.033). There was no difference in tumour size between the two cohorts with both having a median tumour size of 13 cm (Standard range 10–29, ILP range 10–34), (p = 0.915 Mann–Whitney test). The proportion of grade 2 and 3 tumours was also similar with the Standard cohort having 52 (65%) of patients with grade 3 tumours and the ILP cohort 31 patients (70.5%) (p = 0.840 Fisher’s exact test). No significant difference was found in the score of resectability between the two groups (Standard Cohort vs ILP median 4.45 vs 5.05 p = 0.314, mean 5.12 vs 4.23 p = 0.112) although the ILP cohort had slightly higher absolute scores (Additional file 1: Figure S1).

**Table 1:** Patient and tumour characteristics

	Standard cohort	ILP cohort	Overall
Number of patients	80	44	124
Age at operation (median), years	60.5	56	57.5
Gender			
Male	46 (57.5%)	24 (54.5%)	70 (56.5%)
Female	34 (42.5%)	20 (45.5%)	54 (43.5%)
Tumour site			
Thigh	74 (92.5%)	30 (68.2%)	104 (83.9%)
Popliteal fossa	1 (1.3%)	5 (11.4%)	6 (4.8%)
Leg	5 (6.3%)	9 (20.5%)	14 (11.3%)
Median tumour size (cm)	13	13	13
Tumour grade			
II	28 (35.0%)	13 (29.5%)	41 (33.1%)
III	52 (65.0%)	31 (70.5%)	83 (66.9%)
Histology			
Pleomorphic	35 (43.8%)	20 (45.5%)	55 (44.4%)
Liposarcoma	13 (16.3%)	10 (22.7%)	23 (18.5%)
Leiomyosarcoma	9 (11.3%)	5 (11.4%)	14 (11.3%)
Solitary fibrous tumour	3 (3.8%)	1 (2.3%)	4 (3.2%)
MPNST	2 (2.5%)	2 (4.5%)	4 (3.2%)
Other	18 (23.8%)	6 (13.6%)	24 (19.4%)

*MPNST* malignant peripheral nerve sheath tumour

### Treatment

Details of operative characteristics, complications, resection margins, final tumour histology and follow-up are shown in Table 2. Patients in the ILP cohort were significantly more likely to undergo vascular reconstruction. The use of flaps was also more common in this cohort. No significant difference in significant complications, defined as wound infections or collections requiring surgical or radiological intervention, was noted between the two cohorts. Two patients (5%) in the ILP cohort required an amputation. One amputation was performed in the peri-operative period due to procedure-related complications. The other amputation was performed 18 months after surgery due to chronic ulceration following a wound infection. No patients in the Standard cohort required amputation. In the Standard cohort 66 (82%) of patients received adjuvant radiotherapy compared to 12 (27%) patients in the ILP cohort (p < 0.001, Chi square). The reasons for omitting radiotherapy in the Standard cohort were age and associated performance status (4 patients, median age 87.3 years), a compartmental resection achieving negative margins in all planes (6 patients, median age 57.7 years) or the rapid development of pulmonary metastases in the early post-operative period (4 patients). In the ILP cohort, the local recurrence rate in patients not receiving adjuvant radiotherapy was not significantly different to those who did (12.5% vs 8%, p = 1.0 unpaired t-test).

**Table 2:** Post-operative outcomes in standard and ILP cohorts

	Standard N = 80	ILP N = 44	p value
Complications			
All	25 (31%)	17 (39%)	0.43
Wound infection	12 (15%)	7 (16%)	1.0
Collection	13 (16%)	8 (18%)	0.81
Amputation	0 (0%)	2 (5%)	0.12
Resection margin			
R0	65 (81%)	39 (89%)	0.32
R1	15 (19%)	5 (11%)	
Follow-up, months			
Median	31	36	0.3373
(Range)	(4–194)	(8–163)	
Local recurrence	13 (16%)	5 (11%)	0.597
Local recurrence OR post-operative amputation	13 (16%)	7 (16%)	1
Systemic recurrence	43 (54%)	23 (52%)	1

### Oncological Outcomes

**Fig. 3:**
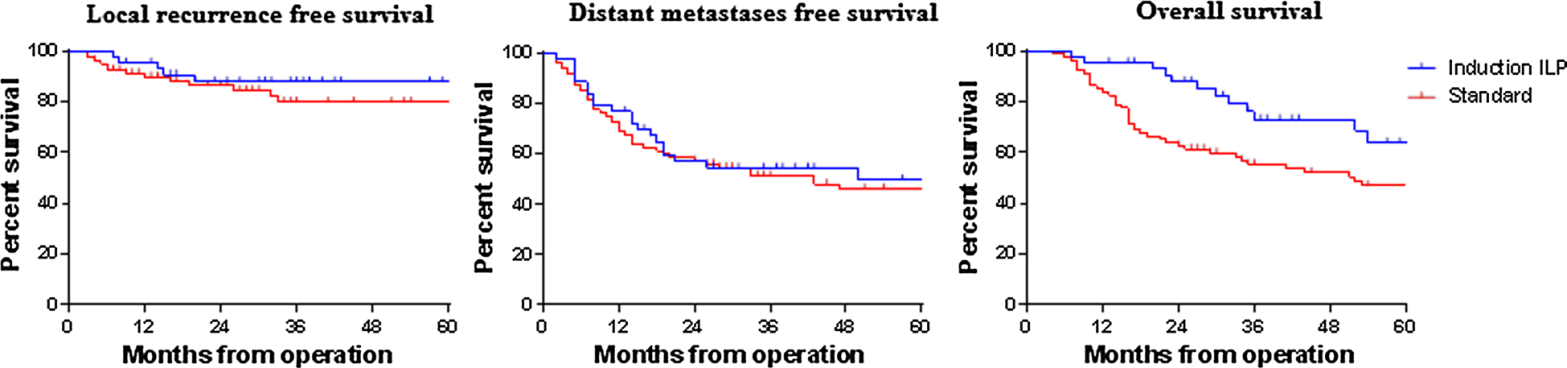
Oncological outcomes for the ILP and Standard cohorts (logrank test was used to assess comparison)

The median follow-up for the Standard and ILP cohorts was 31 (4–194) months and 36 (8–163) months, respectively. The 5-year local recurrence-free (ILP cohort 12.2%, Standard cohort 20.1%, p = 0.375 log-rank test) and distant metastases-free survival (ILP cohort 49.6%, Standard cohort 46.0%, p = 0.821 log-rank test) did not significantly differ between cohorts (Fig. 3). Furthermore, at the time of writing, the local failure rate (i.e. patients developing local recurrence or requiring amputation) for both treatment arms was identical at 16%. Treatment strategy was not predictive of local or distant recurrence, with the only factors found to increase the risk of local recurrence on multivariate analysis being age and a positive (R1) resection margin (Table 3). The 5-year overall survival was worse in the Standard cohort compared to the ILP cohort (46.8% vs 63.8%, p = 0.020 log-rank test). However, in a Cox proportional hazard model, when the differences in age between the two cohorts were accounted for, the overall survival on multivariate analysis between the ILP and Standard cohorts was virtually identical (HR 1.02, 95% CI 1.00–1.04, p = 0.043).

**Table 3:** Multivariate cox proportional hazard analysis of factors effecting survival and recurrence of tumours by cohort

	Local recurrence		Systemic recurrence	
	HR (95% CI)	p value	HR (95% CI)	p value
Standard	1.66 (0.64–4.27)	0.295	1.04 (0.63–1.73)	0.867
ILP	1		1	
Increasing Age	1.04 (1.01–1.08)	0.02	1.02 (0.99–1.03)	0.78
			1	
Increasing size	0.951 (0.84–1.08)	0.44	1.04 (0.99–1.09)	0.98
			1	
R1	3.63 (1.36–9.69)	0.01	1.56 (0.85–2.89)	0.153
R0	1		1	
Grade 3	1.74 (0.56–5.35)	0.33	0.91 (0.55–1.51)	0.704
Grade 2	1		1	

## Discussion

Standard treatment for locally advanced ESTS consists of limb-conserving surgery with adjuvant radiotherapy that may be delivered pre or post-operatively [15]. The role of (neo)adjuvant chemotherapy outside of specific chemo-sensitive subtypes in ESTS is controversial. In the EORTC 62931 study, Woll et al. randomised patients with localised, grade II or III extremity sarcoma to receive adjuvant cheomotherapy in the form of doxorubicin, ifosfamide and lenogastrim in addition to surgery, radiotherapy and, if appropriate, ILP [16]. No benefit in terms of relapse-free or overall survival with this adjuvant regime compared to the control cohort were identified (5-year OS 66.5% vs 67.8%). More recently, in the ISG-STS 1001 study, Gronchi et al. randomised patients with localised, high-grade extremity sarcoma of 5 specified subtypes to receive neoadjuvant standard chemotherapy, in the form of epirubicin and ifosfamide, or histotype-tailored regimes [17]. This trial closed early after an interim analysis demonstrated no benefit in the histotype-tailored regimes. However, at 48 months, overall suvrival in the standard cohort was 89%, which suggests a potential benefit to neoadjuvant standard chemotherapy in these histological subtypes. As of yet, there is consensus regarding the role of (neo)adjuvant chemotherapy and its use is not considered standard in the most recent guidelines [18]. In contrast, induction chemotherapy with ILP is widely recognised to produce markedly better response rates than systemic chemotherapy and has a well-established role in facilitating function-preserving resections in locally advanced ESTS that would otherwise require amputation [7, 19–24].

With induction ILP increasingly used for ESTS that may be amenable to standard surgical management, the question as to which strategy, if either, is superior has arisen. Previous case series have clearly shown that induction ILP prior to surgery is an effective approach to deal with compromised surgical margins, as wide surgical resections were not possible. Similarly adjuvant radiotherapy has been shown to be effective in preventing local relapse after positive surgical margins [25]. The ability to directly compare these treatment strategies is hampered by the rarity of locally advanced non-metastatic primary ESTS and the scarcity of specialist centres performing ILP. As such, a randomized study comparing these strategies is not feasible [26]. Although subject to the limitations of any retrospective study, this comparison of matched cohorts provides valuable evidence to compare these two approaches.

The present study has found that the peri-operative and oncological outcomes of patients undergoing induction ILP prior to surgical resection are very similar to those undergoing standard surgical management. No significant difference was found in the rate of peri-operative complications between cohorts. Although two patients in the ILP cohort required amputations, only one was due to procedure-related complications. Similarly, no significant difference was found in the rates of local recurrence between these treatment arms, despite the use of adjuvant radiotherapy being significantly less frequent in the ILP cohort. There are no clear guidelines regarding the use of radiotherapy following ILP, although it is typically considered following an inadequate response on histological assessment of the specimen. However, following a microscopically complete resection with over 50% necrosis in the specimen, adjuvant radiotherapy is unlikely to be of additional benefit [27].

As a retrospective study, this series is subject to the bias inherent with this methodology. However, analysis of the tumour characteristics in terms of grade, size and histological subtype showed that the cohorts were well matched and this is reflected in the identical rate of metastatic spread. Similarly the assessment of the tumours “resectability” by independent experts indicated that the technical difficulty of surgery appeared to be similar in both cohorts. Although the cohorts were reasonably well matched, they were not randomized and we identified some important differences. There was a higher proportion of popliteal fossa and leg tumours in the ILP cohort. It is generally accepted that achieving local control for sarcomas is more challenging in the distal than the proximal extremity. Therefore, this may represent a bias towards patients with more challenging tumours being referred to specialist centres providing ILP. A further limitation to this study is the inability to comment on the post-operative limb-function in each cohort, an additional important factor that may influence the choice of treatment strategy in these patients.

In the absence of any significant difference in outcomes between these treatment strategies, the morbidity associated with their use becomes increasingly important. Induction ILP carries the risks associated with an additional operation and short term toxicity associated with regional chemotherapy. That being said, the peri-operative morbidity associated with ILP is generally very low. Severe regional toxicity following ILP is rare occurring in 2–15% and the need for amputation even more so with rates of 0–2% [28]. The long-term complications from external beam radiotherapy, the technique used in the majority of patients in this study, are well documented [29]. However, during the period of this study, advances have been made in radiotherapy techniques. When given pre-operatively, the dose of radiotherapy may be reduced and the use of intensity-modulated radiotherapy, which is associated with lower toxicity rates, is becoming more widespread [15, 30]. Even so, the long-term sequelae of radiotherapy, including the risk of second malignancies, remain significant and are of particular importance in young patients [31].

## Conclusion

Induction ILP followed by a wide resection provides an alternative treatment to standard therapy in the management of locally advanced ESTS. Consideration should be given to induction ILP in young patients with large, high-grade extremity sarcomas who would benefit most from avoiding radiotherapy.

## Additional file

**Additional file 1: Figure S1.** Scatter plot of individual tumour ratings and median scores of resectability by cohort as assessed by MRI imaging (see Fig. 1) (statistical analysis was performed using Mann–Whitney test).
