# Supplementary figures and images for: Regional chemotherapy by isolated limb perfusion prior to surgery compared with surgery and post-operative radiotherapy for primary, locally advanced extremity sarcoma: a comparison of matched cohorts
Source: Clin Sarcoma Res. 2018 Jul 2;8:12. doi: 10.1186/s13569-018-0098-6 (PMC6027577; doi:10.1186/s13569-018-0098-6)

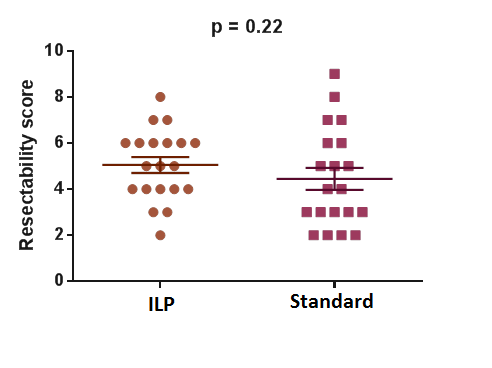

Supplement: Supplementary file 1 — Additional file 1: Figure S1. Scatter plot of individual tumour ratings and median scores of resectability by cohort as assessed by MRI imaging (see Fig. 1) (statistical analysis was performed using Mann–Whitney test). [file 13569_2018_98_MOESM1_ESM.tif]
